# Blood Metabolites Mediate the Effects of Gut Microbiota on Diabetic Nephropathy: A Mendelian Randomization Study

**DOI:** 10.1155/ije/6642332

**Published:** 2026-07-14

**Authors:** Weiwei Zhang, Yun Zhang, Huijuan Yuan

**Affiliations:** ^1^ Department of Endocrinology, Henan Provincial People’s Hospital, Xinxiang Medical University, Xinxiang, Henan, China, xxmu.edu.cn; ^2^ Department of Endocrinology, Henan Provincial People’s Hospital, Zhengzhou, Henan, China, hnsrmyy.net

**Keywords:** blood metabolomes, diabetic nephropathy, gut microbiota, immune cells, Mendelian randomization, plasma lipidomes

## Abstract

**Background:**

The gut microbiota (GM) might be related to diabetic nephropathy (DN). The role of blood metabolites, immune cells, and plasma lipidomes in mediating the impact of GM on DN remains unexplored.

**Methods:**

412 GM, 1400 blood metabolites, 731 immune cells, 179 plasma lipidomes, and DN were identified through genome‐wide association study (GWAS) datasets. We employed Mendelian randomization (MR) to investigate the relationship among GM, blood metabolomes, immune cells, plasma lipidomes, and DN. Furthermore, we conducted mediation analyses to ascertain if the blood metabolomes, immune cells, and plasma lipidomes function as mediators.

**Results:**

Our research identified suggestive causal linkages among 15 GM, 27 immune cells, 27 plasma lipidomes, and 55 blood metabolites associated with DN. Mediation analysis showed that advanced glycation end products (AGEs) pyrraline may mediate the causal relationship between s_Bacteroides_eggerthii and DN, with a mediation percentage of 39.96% (*p* = 0.015). Furthermore, immune cells and plasma lipidomes do not appear to play a mediating role.

**Conclusion:**

Gut microbiota, blood metabolomes, immune cells, and plasma lipidomes were causally linked to DN. The blood metabolite pyrraline may act as a potential mediator by which s_Bacteroides_eggerthii influences the risk of developing DN.

## 1. Introduction

Diabetic nephropathy is a serious microvascular complication of diabetes mellitus, affecting around 30%–40% of diabetic patients and serving as the primary cause of end‐stage renal disease (ESRD) [1, 2]. The main driver of diabetic kidney disease is chronic hyperglycemia. The onset of hyperglycemia initiates a series of pathophysiological disturbances, such as altered tubuloglomerular feedback, renal hypoxia, hypertension, lipotoxicity, podocyte injury, inflammation, mitochondrial dysfunction, impaired autophagy, and heightened sodium–hydrogen exchanger activity. These abnormalities collectively result in progressive glomerular sclerosis and a reduction in glomerular filtration rate [3–5]. Nonetheless, contemporary multimodal intervention strategies aimed at mitigating the risk of diabetic nephropathy (DN) have proven insufficient, mainly attributable to the absence of treatment that effectively and specifically targets the molecular characteristics of DN. Therefore, we are specifically targeting patients with DN to further clarify the mechanisms of renal fibrosis in DN and to discover novel biomarkers or targets linked to the ongoing deterioration of renal function in these patients.

The number of bacterial cells living in our bodies and on the surface of our bodies is about 38 trillion, and the number of human cells in our bodies is about 30 trillion [6, 7]. Gut microbiota and host interactions [8]. The gut microbiota may affect DN via regulating blood concentrations of specific physiologically active metabolites (e.g., trimethylamine N‐oxide and short‐chain fatty acids) and the immune system [9, 10]. Consequently, we hypothesized a potential causal connection between gut microbiota, blood metabolites, immune cells, plasma lipidomes, and DN. We hypothesize that blood metabolites, immune cells, and plasma lipidome might function as mediators between gut microbiota and DN. We aimed to clarify these connections and discover potential metabolites for early diagnosis and clinical treatment targets.

Mendelian randomization (MR) employs single nucleotide polymorphisms (SNPs) as instrumental variables (IVs) for inferring causal links between exposures and outcomes in genetic research [11]. MR can simulate randomized controlled trials with random assignment of SNPs, eliminating bias from reverse causality and reducing confounding commonly associated with traditional epidemiological studies [12, 13]. We performed an extensive MR analysis to investigate causality between gut microbiota, blood metabolites, immune cells, plasma lipidomes, and DN. We conducted mediation analyses to ascertain if blood metabolites, immune cells, and plasma lipidomes could mediate a causal link among gut microbiota with DN. This study primarily focuses on blood metabolites, while immune cell and plasma lipidome analyses are secondary objectives.

## 2. Methods

### 2.1. Study Design

**FIGURE 1:**
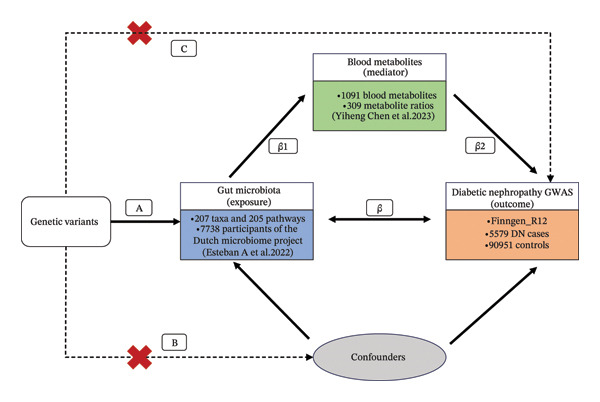
Study design overview. (A) Relevance assumption. (B) Independence assumption. (C) Exclusivity assumption. (β) Gut microbiota–diabetic nephropathy. (β1) Gut microbiota–blood metabolites. (β2) Blood metabolites–diabetic nephropathy.

This study adhered to the reporting guidelines of MR research (STROBE‐MR) [14, 15] (Additional file 3). MR depends upon three core principles: (A) the hypothesis of relevance: IVs must exhibit a strong association to exposures (gut microbiota, blood metabolomes, immune cells, and plasma lipidomes); (B) the hypothesis of independence: To assess the correlation of linkage disequilibrium (LD) between SNPs strongly associated with exposure, LD < 0.001, and IVs are unassociated with confounding factors; and (C) the hypothesis of exclusion: IVs affect the outcome only through their effect on exposure, horizontal pleiotropy *p* > 0.05 [16]. Figure 1 displays the research graph.

### 2.2. Data Source

Data on 412 gut microbiomes were newly acquired from a study conducted by Esteban et al. that included 7738 participants [17]. We acquired 1091 blood metabolites and 309 metabolite ratios from 8299 participants in the Canadian Longitudinal Study of Aging (CLSA) cohort, as reported by Yiheng Chen et al. Among these, 850 metabolites exhibited established characteristics throughout 8 major pathways, including lipids, amino acids, exogenous substances, nucleotides, cofactors and vitamins, carbohydrates, peptides, and energy. The remaining 241 were categorized as unknown or “partially” described molecules [18]. The GWAS data for 731 immune cells were sourced from a study that examined these characteristics in a cohort of 3757 Sardinian individuals [19]. We obtained 179 plasma lipid groups from Ottensmann et al., whose study comprised 7174 participants [20]. We obtained the available GWAS data on DN through the latest FinnGen R12 database. Additional file 1: Comprehensive data source description is presented in Table S1. Our work did not need extra ethical review, as the original GWAS study had ethical approval.

### 2.3. Data Extraction

Based on previous MR studies [21–25], in order to identify SNPs as the IVs linked to gut microbiota, 1400 blood metabolomes, 731 immune cells, and 179 plasma lipidomes, we first set the significance criterion of *P*_1_ < 5 × 10^−8^. The greater permissive criterion with *P*_1_ < 1 × 10^−5^ used to identify SNPs associated with gut microbiota because there were only a few SNPs linked to gut microbiota, blood metabolomes, immune cells, and plasma lipidomes found at the *P*_1_ < 5 × 10^−8^ threshold. To identify SNPs associated with immune cells, plasma lipidomes, and blood metabolites, a threshold of *P*_1_ < 5 × 10^−6^ was selected. Second, an LD analysis was conducted on the SNPs to ensure independence between SNPs with a threshold of *r* ^2^ < 0.001 and a distance of 10,000 kb. Third, SNPs with *P*_2_ < 5e‐5 were filtered for correlation with the outcome [26]. To ensure the rigor and robustness of our research findings, we exercise strict control over the quality of our IVs. SNPs were filtered based on instrumental strength, which was quantified by genetic variability (*R* ^2^) and F‐statistic; those with an F‐statistic below 10 were excluded [27]. *R* ^2^ = 2 × (1‐MAF) × MAF × β [2] (where MAF is the minor allele frequency and β is the effect size of the SNP used as the IV for the exposure), *F* = [*R* ^2^/(1 − *R* ^2^)] × [(N‐k‐1)/*k*)](*N* is the total sample size, and *k* is the number of IVs) [28]. Additionally, the robust adjusted profile score (RAPS) provides robust inference under a framework that accommodates weak instruments [29]. The debiased inverse variance weighted (DIVW) method specifically corrects for the weak‐instrument bias of the IVW method [30]. Detailed descriptions of the DIVW and RAPS methods are provided in the MR analysis section. Therefore, we employ RAPS and DIVW to further mitigate the bias arising from weak instruments. LDlinkR was used to identify SNPs that were associated with potential confounders (body mass index, smoking, alcohol use, etc.). SNPs showing such associations at the genome‐wide significance threshold were excluded. This prevents these SNPs from interfering with the effect of the exposure on the outcome.

### 2.4. MR Analysis

To estimate the causal impact of gut microbiota, blood metabolomes, immune cells, and plasma lipidomes on DN, we conducted a two‐sample MR study. When horizontal pleiotropy is balanced, the IVW method is used as the main analytical approach. The IVW method is a meta‐analytic weighted average of the ratio estimates, employing the reciprocal of their approximate variance as weights [31, 32]. MR‐Egger, weighted median (WM), weighted mode, DIVW, RAPS, and the contamination mixture method (ConMix) as supplementary methods. MR‐Egger does not constrain the regression line to intersect the origin, thereby accommodating directed genetic pleiotropy within the IVs utilized. The distinction between MR‐Egger and IVW lies in the incorporation of the intercept term in the regression analysis [33]. The WM ranks individual SNP effect sizes in order of magnitude, cumulates their weights, and takes the effect size corresponding to a cumulative weight of 50% as the estimate; when at least 50% of the weights come from valid IVs, the estimate remains consistent and robust even if half of the weights are invalid [34]. The weighted mode method produces a pleiotropy‐robust estimate, assuming that the most common variant‐specific effect remains consistent despite the presence of invalid instruments [35]. The DIVW aims to eradicate the weak instrumental bias inherent in the IVW technique, demonstrating enhanced robustness in the presence of numerous weak IVs [30]. The RAPS method accommodates weak instruments, through which robust MR estimates can be obtained [29]. The advantage of the ConMix is its ability to accommodate hundreds of IVs while still providing reliable causal estimates in the presence of invalid IVs [36].

We performed sensitivity analyses to assess result robustness. Heterogeneity among the chosen genetic variants was examined using Cochran’s *Q* test, with a *p* value below 0.05 defined as significant heterogeneity [32]. Potential horizontal pleiotropy was evaluated using the MR‐Egger intercept along with the MR‐PRESSO global test [33, 37]. We carried out a leave‐one‐out analysis to assess the robustness of the causal estimates and to gauge the influence of each individual SNP on the outcomes [38].

### 2.5. Mediation Analysis

The assumption underlying MR‐based mediation is a linear relationship, no exposure–mediator interaction, and no mediator–outcome pleiotropy [39]. We further search for potential mediators from blood metabolomics, immune cells, and plasma lipidomes. First, we investigated the causal link between the gut microbiome and DN and then identified blood metabolomics, immune cells, and plasma lipidome groups that were significantly and causally associated with DN and did not show heterogeneity or pleiotropy. Then, we performed a Mendelian analysis of the gut microbiota on blood metabolomics, immune cells, and plasma lipidomes. The direction of the effect value should be as follows: if β is positive, then both β1 and β2 are either positive or negative. Conversely, if β is negative, then either β1 or β2 should be positive, while the other is negative (Figure 1). Finally, the mediation effect was calculated as the product of the coefficients. We then divided this effect by the total effect to obtain the proportion mediated. The 95% confidence interval (CI) of the mediation effect was computed via the Delta method [40]. The results need to fulfill *p* value < 0.05. The proportion of mediation and its confidence interval must be positive.

All MR analyses were performed in the R software (V.4.4.0) (https://www.R-project.org), using the “TwoSampleMR (Version 0.6.8)”, “Rmediation 1.2.2”, “MRPRESSO package (1.0)”, “MendelianRandomization (Version 0.8.0)” [41, 42], and the “LdlinkR” package [43, 44]. The FDR *q* value is estimated using the R package “*p.adjust*” [45].

## 3. Results

### 3.1. The Selection of IVs

A total of 3914 SNPs related to the gut microbiota were selected after LD clumping (LD, threshold set to 10,000 kb, *r* ^2^ < 0.001) and F‐statistics exceeding 10, based on a *p* value of 1 × 10^−5^ (Additional file 1: Table S2). In addition to LD clumping and F‐statistics above 10. Under a threshold of *P*1 < 5 × 10^−6^, we identified 19,145, 9935, and 2732 SNPs associated with blood metabolites, immune cells, and plasma lipidomes (Additional file 1: Tables S3–S5).

### 3.2. Causal Effects of Gut Microbiota on DN

**FIGURE 2:**
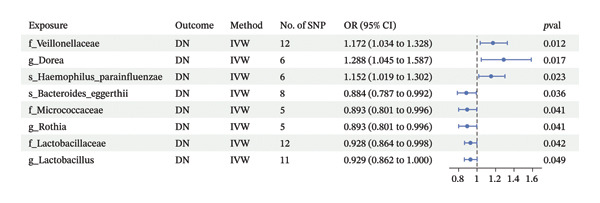
Forest plot of results for 8 GM taxa (f = families, g = genera, and s = species) causally associated with DN. OR: odds ratio; CI: confidence interval. DN: diabetic nephropathy; IVW: inverse variance weighted.

**FIGURE 3:**
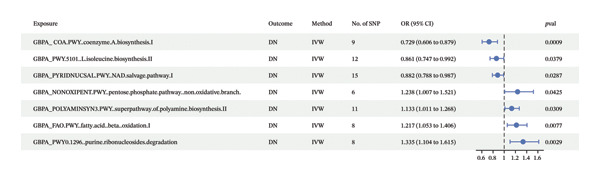
Forest plot of results for 7 gut bacterial pathway abundances (GBPAs) causally associated with DN. OR: odds ratio; CI: confidence interval; DN: diabetic nephropathy; IVW: inverse variance weighted.

We conducted an MR investigation between gut microbiota and DN (Additional file 1: Tables S6 and S18). Utilizing the IVW estimate procedure (*p* < 0.05), alongside seven additional methodologies, we ultimately uncovered 15 causal correlations between gut microbiota and DN[8 gut microbiota taxa (4 families, 2 genera, and 2 species from p_Firmicutes, p_Proteobacteria, p_Bacteroidetes, and p_Actinobacteria) and 7 GBPAs]. Among the gut microbiota taxa, s_Bacteroides_eggerthii (or = 0.884, 95% CI = 0.787–0.992, *p* = 0.036) was associated with a reduced risk of developing DN, and g_Dorea (or = 1.288, 95% CI = 1.045–1.587, *p* = 0.017) was associated with an increased risk of developing DN. GBPA_COA.PWY (or = 0.729, 95% CI = 0.606–0.879, *p* = 0.0009) was associated with a reduced risk of developing DN in GBPAs. GBPA_PWY0.1296 (or = 1.335, 95% CI = 1.104–1.615, *p* = 0.0029) was associated with an increased risk of developing DN. All results showed suggestive correlations after FDR correction (Additional file 1: Table S6, Figures 2 and 3).

### 3.3. Causal Effects of the Blood Metabolomes on DN

**FIGURE 4:**
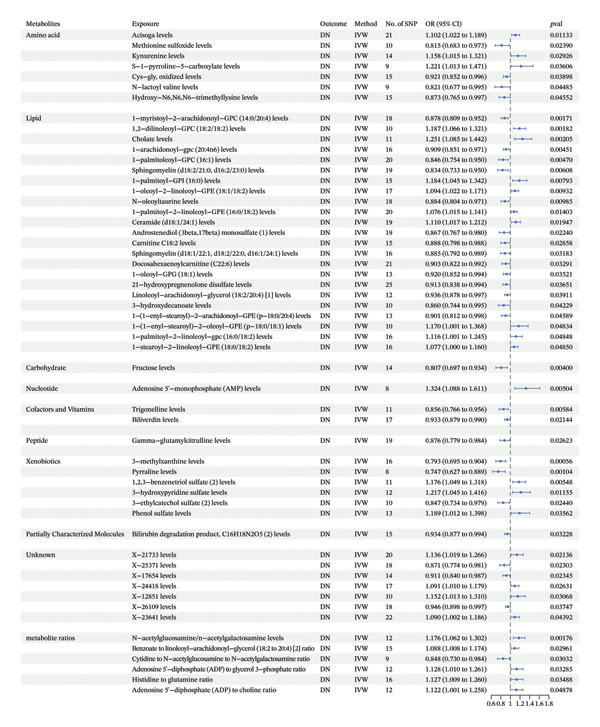
Forest plot of results for 55 blood metabolites causally associated with DN. OR: odds ratio; CI: confidence interval; DN: diabetic nephropathy; IVW: inverse variance weighted.

We then discovered 55 significant links between blood metabolomes and DN (7 amino acids, 23 lipids, 1 carbohydrate, 1 nucleotide, 2 cofactors and vitamins, 1 peptide, 6 xenobiotics, 1 partially characterized molecule, 7 unknowns, and 6 metabolite ratios). Among the amino acids, methionine sulfoxide (or = 0.815, 95% CI = 0.683–0.973, *p* = 0.024) was associated with a reduced risk of DN. S‐1‐pyrroline‐5‐carboxylate (or = 1.221, 95% CI = 1.013–1.471, *p* = 0.036) was associated with an increased risk of DN. In lipid, sphingomyelin (or = 0.834, 95% CI = 0.733–0.949, *p* = 0.006) was associated with a reduced risk of developing DN, and cholate (or = 1.250, 95% CI = 1.084–1.441, *p* = 0.002) was associated with an increased risk of developing DN. Fructose (or = 0.807, 95% CI = 0.697–0.934, *p* = 0.004) was associated with a reduced risk of DN in carbohydrates. Adenosine 5′‐monophosphate (or = 1.324, 95% CI = 1.088–1.611, *p* = 0.005) was associated with an increased risk of DN among the nucleotides. Trigonelline (or = 0.856, 95% CI = 0.766–0.956, *p* = 0.006) was associated with reduced risk of DN in Coactors and Vitamins. Gamma‐glutamylcitrulline is a peptide (or = 0.876, 95% CI = 0.779–0.984, *p* = 0.026) that is associated with a reduced risk of DN. In Xenobiotics, pyrraline (or = 0.747, 95% CI = 0.627–0.889, *p* = 0.001) was associated with a reduced risk of DN, and 3‐hydroxypyridine sulfate (or = 1.217, 95% CI = 1.045–1.416, *p* = 0.012) was associated with an increased risk of DN. In partially characterized molecules, the bilirubin degradation product C16H18N2O5 (or = 0.934, 95% CI = 0.877–0.994, *p* = 0.032) was associated with a reduced risk of DN. Cytidine to N‐acetylglucosamine to N‐acetylgalactosamine (or = 0.848, 95% CI = 0.730–0.984, *p* = 0.030) was associated with a reduced risk of DN in the metabolite ratios. N‐acetylglucosamine/n‐acetylgalactosamine (or = 1.176, 95% CI = 1.062–1.302, *p* = 0.002) was associated with an increased risk of developing DN (Additional file 1: Table S7, Figure 4).

### 3.4. Causal Effects of Immune Cells and Plasma Lipidomes on DN

**FIGURE 5:**
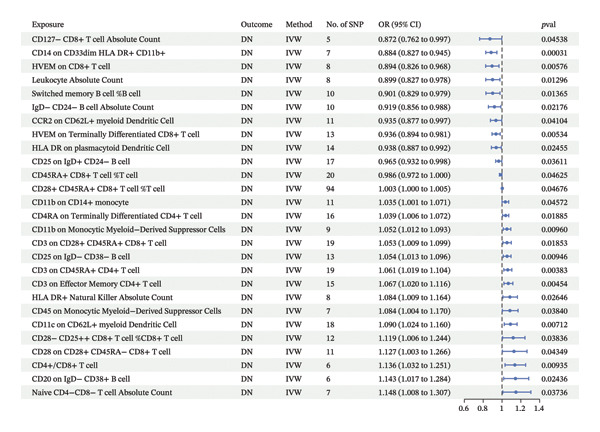
Forest plot of results for 27 immune cells causally associated with DN; OR: odds ratio; CI: confidence interval; DN: diabetic nephropathy; IVW: inverse variance weighted.

**FIGURE 6:**
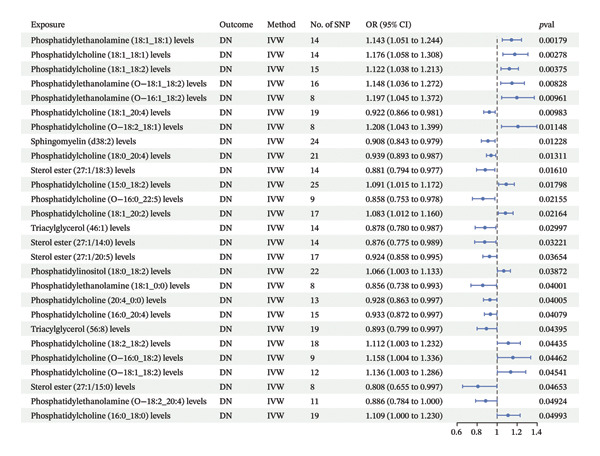
Forest plot of results for 27 plasma lipidomes causally associated with DN; OR: odds ratio; CI: confidence interval; DN: diabetic nephropathy; IVW: inverse variance weighted.

Twenty‐seven pairs of immune cells containing 11 protective factors and 16 risk factors were identified. CD127‐CD8+ T cell absolute count (or = 0.872, 95% CI = 0.762–0.997, *p* = 0.045) was associated with a reduced risk of developing DN; Naive CD4‐CD8‐T cell absolute count (or = 1.148, 95% CI = 1.008–1.307, *p* = 0.037) was associated with an increased risk of DN (Additional file 1: Table S8, Figure 5). Twenty‐seven pairs of plasma lipidomes containing 14 protective factors and 13 risk factors were identified. Sterol ester (or = 0.808, 95% CI = 0.655–0.997, *p* = 0.047) was associated with a reduced risk of DN, and phosphatidylcholine (or = 1.208, 95% CI = 1.043–1.399, *p* = 0.011) was associated with an increased risk of DN (Additional file 1: Table S9, Figure 6).

### 3.5. Sensitivity Analysis

The Cochran *Q* test in sensitivity analysis indicated an absence of heterogeneity in the relationship between gut microbiota and DN. Both the MR‐Egger intercept and MR‐PRESSO tests indicated an absence of horizontal pleiotropy (*p* > 0.05, Additional file 1: Tables S10 and S11). The Cochran *Q* test indicated an absence of heterogeneity in the relationships between blood metabolomics groups and DN, while the MR‐PRESSO test revealed no horizontal pleiotropy. The MR‐Egger intercept test revealed no horizontal pleiotropy, with the exceptions of threonate, 5‐dodecenoylcarnitine, and pyruvate to 3‐methyl‐2‐oxobutyrate. (*p* > 0.05, Additional file 1: Tables S12 and S13). These three data have been excluded. Immune cells and plasma lipidomes showed no heterogeneity in the Cochran *Q* test, no horizontal pleiotropy in the MR‐PRESSO test, and no horizontal pleiotropy in the MR‐Egger intercept test. (*p* > 0.05, Additional file 1: Tables S14–S17). The leave‐one‐out analysis indicated that no one SNP significantly influenced the causal relationship between gut microbiota and DN (Additional file 2: Figures S1 and S2).

### 3.6. Mediation Analysis

**FIGURE 7:**
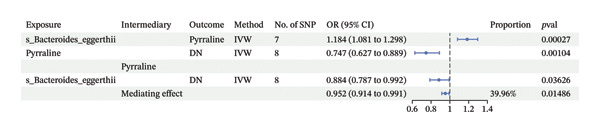
The mediating role of pyrraline in the causal relationship between s_Bacteroides_eggerthii and DN. OR: odds ratio; CI: confidence interval. DN: diabetic nephropathy; IVW: inverse variance weighted.

**FIGURE 8:**
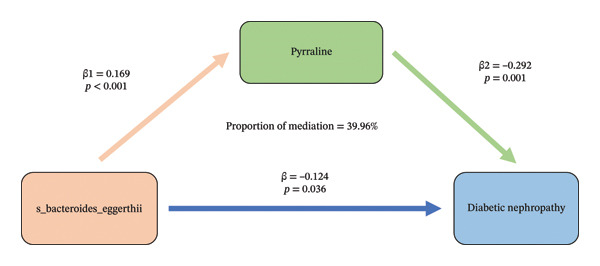
The mediating role of pyrraline in the causal relationship between s_Bacteroides_eggerthii and diabetic nephropathy.

Batch Mendelian analysis of gut microbiota and blood metabolomes at *p* < 0.05 was then performed, and 42 potential gut microbiota‐blood metabolomes‐DN pathways were initially identified (Table S19). Neither mediation to outcome, exposure to mediation, nor exposure to outcome showed horizontal pleiotropy or heterogeneity. The mediating effect (or = 0.952, 95% CI = 0.914–0.991, *p* = 0.015) was calculated by the product of coefficients approach, which accounted for 39.96% of the total effect. A causal pathway between s_Bacteroides_eggerthii and DN mediated by pyrraline was finally identified (Table S22, Figure 7). The effect size β1 of s_Bacteroides_eggerthii on pyrraline is 0.169. The effect size β2 of pyrraline on DN is −0.292. The effect size β of s_Bacteroides_eggerthii on DN is −0.124 (Figure 8). However, pyrraline is shown here as a protective factor for DN. This may be because MR reflects lifetime genetic exposure rather than the circulating burden of AGEs. Similarly, gut microbiota‐immune cells‐DN and gut microbiota‐plasma lipidomes‐DN were validated, but no mediating effect appeared to be present.

## 4. Discussion

To assess the potential causal association between gut microbiota and DN, we applied a bidirectional two‐sample MR approach. The findings showed a causal association between eight gut microbiota and seven GBPAs with DN. There were 55 causal associations between the blood metabolomes and DN, 27 causal relationships among immune cells and DN, and 27 causal associations between the plasma lipidomes and DN in the unidirectional two‐sample MR, but only in the blood metabolomes was pyrraline found to mediate a causal pathway between s_Bacteroides_eggerthii and DN.

In our study, s_Bacteroides_eggerthii was associated with the greatest reduction in the risk of developing DN. Research has demonstrated that s_Bacteroides_eggerthii exhibits a strong correlation with the stage of chronic kidney disease (CKD) and is valuable in early diagnosis, with possible etiologic and diagnostic implications [46, 47]. This further corroborates our results. Among other protective factors, a prospective cohort study by Gan et al. suggests that increased exposure to air pollutants may affect adverse pregnancy outcomes by altering the abundance of f_Micrococcaceae and g_Rothia [48]. A clinical investigation conducted by Soleimani et al. exhibits advantageous impacts of f_Lactobacillaceae_g_Lactobacillus supplementation throughout a 12 week period in diabetic hemodialysis patients regarding glucose homeostasis and other indicators of inflammation and oxidative stress [49]. Yi‐Shen‐Hua‐Shi granule, a combination of Astragalus and Salvia miltiorrhiza, and Astragalus polysaccharide have demonstrated a considerable rise in the abundance of f_Lactobacillaceae_g_Lactobacillus in a number of animal experiments to ameliorate DN via the gut‐renal axis pathway [50–52]. In risk factors, an acidic polysaccharide extracted from the cortex of Eucommia ulmoides Oliver modifies g_Dorea and mitigates oxidative stress in animal tests conducted by Song, et al. [53]. Elevated f_veillonellaceae abundance is correlated with increased nonalcoholic fatty liver disease (NAFLD) risk in animal experiments by Zeng et al. [54]. Barberio, et al. Higher levels of s_Haemophilus_parainfluenzae were found in ulcerative colitis in machine learning [55]. A clinical study by Saulnier et al. demonstrates a significant increase in s_Haemophilus_parainfluenzae in children with irritable bowel syndrome [56]. Our study identified some GBPAs as causally linked to DN. Metabolites of gut microbiota generated by GBPAs can influence DN through the gut‐renal axis pathway [9].

To investigate the particular pathways via which gut microbiota affect DN, the critical role of the blood metabolomes as a mediator was further analyzed. Our analysis found a more significant causal relationship between s_Bacteroides_eggerthii, pyrraline, and DN. Pyrraline is a structural form of advanced glycation end products (AGEs), which increase slowly in the tissues of nondiabetic subjects but accumulate in large amounts in diabetic patients as a result of accelerated AGEs production due to persistently high circulating glucose levels. Excess AGEs not only cross‐link with proteins and affect their properties but also bind to specific receptors and react to alter cellular functions, leading to pathological changes in the body. AGEs are intricately linked to the progression of DN [57, 58]. K. C. et al. demonstrated through animal tests that glucagon‐like peptide‐1 receptor signaling influences the severity of diabetic kidney disease by attenuating inflammation generated by the receptor for AGEs (RAGEs) [59].

Serum or plasma levels of AGEs show poor correlation with cardiovascular events in patients with CKD, which is possibly because serum concentrations do not correlate with AGEs deposition in target tissues [60]. The key factor determining its therapeutic efficacy lies in its ability to reduce the glycation burden on tissues [61]. Research confirms that tissue AGEs accumulation assessed by skin autofluorescence is independently associated with DN [62]. Plasma pentosidine concentrations in individuals with DN are affected by factors including renal function and patient age, with renal function being the decisive factor [63]. In chronic renal failure, elevated circulating AGEs levels greater than circulating levels of AGEs arise from enhanced production and reduced clearance [64]. MR capitalizes on the fact that genotypes are typically resistant to reverse causation [65]. The formation of pyrraline may represent a compensatory mechanism in the body under prolonged hyperglycemic stress [66]. Neither NFκB activation nor elevated RAGE expression in the presence of glycated caseins. Conversely, pyrraline‐modified casein exhibits an Nrf2‐dependent antioxidative effect [67]. Therefore, pyrraline served solely as a hypothesis generator in this study, not as a clinical protective agent. We hypothesized that pyrraline mediates the effect of s_Bacteroides_eggerthii on DN. Furthermore, we examined the possible mediating function of immune cells and plasma lipidomes in DN. Consequently, we enhanced our comprehension of the fundamental mechanisms linked to DN.

Strengths of our research include utilizing the latest GWAS data on gut microbiota, blood metabolomics, immune cells, plasma lipidomes, and DN [17–20]. Of course, our study has some limitations. Gut microbiota exhibit variations among specific populations. Existing research indicates significant differences in gut microbiota diversity, abundance, and core strains across different geographic regions [68].Second, metabolomic variability among different ancestral groups. Given that our study participants were predominantly of European ancestry, the applicability of these results to other ethnic or racial groups may be limited and warrants cautious interpretation. In addition, we presumed a linear exposure–outcome association in the MR analysis that might fail to adequately represent connections that could be more intricate and nonlinear. The goal of the current investigation is to produce new hypotheses rather than conclusive findings. As a result, our results ought to be regarded as preliminary and require confirmation in further studies.

## 5. Conclusion

The relationship between DN, immune cells, blood metabolomes, gut microbiota, and plasma lipidomes was examined thoroughly in this study. There are 55 blood metabolites, 27 immune cells, 27 plasma lipidomes, and 15 gut microbiota that are suggestively causally linked to DN. Furthermore, the blood metabolomes may act as a potential mediator in the pathway by which gut microbiota influence the risk of developing DN, whereas immune cells and plasma lipidomes do not fulfill a mediating function. Collectively, these data provide novel clues into the pathogenesis and management of DN.

## Author Contributions

W.Z. and H.Y. conceived and designed the study, W.Z. performed the data analysis and interpreted the results. W.Z. created and optimized the visualizations. W.Z. wrote, revised, and edited the manuscript. Y.Z. and H.Y. wrote–reviewed, edited, and conceptualization. Y.Z. project management, H.Y. funding acquisition.

## Funding

This work was supported by the National Natural Science Foundation of China (82270865), Henan Provincial Key Research and Development Projects (231111313200), and Henan Provincial Medical Science and Technology Research Program‐The Provincial and Ministerial Major Projects (SBGJ202301002).

## Disclosure

All authors read and approved the final version.

## Conflicts of Interest

The authors declare no conflicts of interest.

## Supporting Information

Additional supporting information can be found online in the Supporting Information section.

## Supporting information


**Supporting Information 1** Additional File 1 22 Tables: Table S1: Data sources of GWAS summary obtained for MR analyses. Table S2: Summary of 3914 SNPs significantly associated with the 412 gut microbiota. Table S3: Summary of 19,145 SNPs significantly associated with the 1400 blood metabolites. Table S4: Summary of 9935 SNPs significantly associated with the 731 immune cells. Table S5: Summary of 2732 SNPs significantly associated with the 179 plasma lipidomes. Table S6: Significant causal associations between gut microbiota and diabetic nephropathy. Table S7: Significant causal associations between blood metabolites and diabetic nephropathy. Table S8: Significant causal associations between immune cells and diabetic nephropathy. Table S9: Significant causal associations between plasma lipidomes and diabetic nephropathy. Table S10: Heterogeneity tests between gut microbiota and diabetic nephropathy. Table S11: Pleiotropy tests between gut microbiota and diabetic nephropathy. Table S12: Heterogeneity tests between blood metabolites and diabetic nephropathy. Table S13: Pleiotropy tests between blood metabolites and diabetic nephropathy. Table S14: Heterogeneity tests between immune cells and diabetic nephropathy. Table S15: Pleiotropy tests between immune cells and diabetic nephropathy. Table S16: Heterogeneity tests between plasma lipidomes and diabetic nephropathy. Table S17: Pleiotropy tests between plasma lipidomes and diabetic nephropathy. Table S18: MR results of diabetic nephropathy on gut microbiota. Table S19: IVW results of gut microbiota on blood metabolites associated with diabetic nephropathy. Table S20. IVW results of gut microbiota on immune cells associated with diabetic nephropathy. Table S21: IVW results of gut microbiota on plasma lipidomes associated with diabetic nephropathy. Table S22: Mediation analysis.

**Supporting Information 2** Additional File 2: Figure S1: Leave‐one‐out analysis for gut microbiota on diabetic nephropathy. The analyses of (A) f_Micrococcaceae, (B) f_Lactobacillaceae, (C) f_Veillonellaceae, (D) g_Rothia, (E) g_Lactobacillus, (F) g_Dorea, (G) s_Haemophilus_parainfluenzae, and (H) s_Bacteroides_eggerthii. Figure S2: Leave‐one‐out analysis for gut bacterial pathway abundances (GBPAs) on diabetic nephropathy. The analyses of (A) GBPA_ COA.PWY.coenzyme.A.biosynthesis.I, (B) GBPA_FAO.PWY.fatty.acid.beta.oxidation.I, (C) GBPA_NONOXIPENT.PWY.pentose.phosphate.pathway.non.oxidative.branch., (D) GBPA_POLYAMINSYN3.PWY.superpathway.of.polyamine.biosynthesis.II, (E) GBPA_PWY0.1296.purine.ribonucleosides.degradation, (F) GBPA_PWY.5101.L.isoleucine.biosynthesis.II, and (G) GBPA_PYRIDNUCSAL.PWY.NAD.salvage.pathway.I.

**Supporting Information 3** Additional File 3: STROBE‐MR‐checklist.

## Data Availability

This study’s GWAS summary statistics are derived from publicly accessible datasets, including FinnGen (https://www.finngen.fi/en), IEU Open GWAS (https://gwas.mrcieu.ac.uk/), and GWAS Catalog (https://www.ebi.ac.uk/gwas/). Comprehensive details regarding the data utilized in the studies are available in the supplemental information files.
