# Supplementary material for: Blood Metabolites Mediate the Effects of Gut Microbiota on Diabetic Nephropathy: A Mendelian Randomization Study
Source: Int J Endocrinol. 2026 Jul 14;2026:6642332. doi: 10.1155/ije/6642332 (PMC13369588; doi:10.1155/ije/6642332)
Supplement: Supplementary file 2 — Supporting Information 2 Additional File 2: Figure S1: Leave‐one‐out analysis for gut microbiota on diabetic nephropathy. The analyses of (A) f_Micrococcaceae, (B) f_Lactobacillaceae, (C) f_Veillonellaceae, (D) g_Rothia, (E) g_Lactobacillus, (F) g_Dorea, (G) s_Haemophilus_parainfluenzae, and (H) s_Bacteroides_eggerthii. Figure S2: Leave‐one‐out analysis for gut bacterial pathway abundances (GBPAs) on diabetic nephropathy. The analyses of (A) GBPA_ COA.PWY.coenzyme.A.biosynthesis.I, (B) GBPA_FAO.PWY.fatty.acid.beta.oxidation.I, (C) GBPA_NONOXIPENT.PWY.pentose.phosphate.pathway.non.oxidative.branch., (D) GBPA_POLYAMINSYN3.PWY.superpathway.of.polyamine.biosynthesis.II, (E) GBPA_PWY0.1296.purine.ribonucleosides.degradation, (F) GBPA_PWY.5101.L.isoleucine.biosynthesis.II, and (G) GBPA_PYRIDNUCSAL.PWY.NAD.salvage.pathway.I. [file IJE-2026-6642332-s001.docx]

**Additional file 2. Figures**

**Figure S1.** Leave-one-out analysis for gut microbiota on diabetic nephropathy.

**Figure S2.** Leave-one-out analysis for gut bacterial pathway abundances (GBPAs) on diabetic nephropathy.

**A** **B**


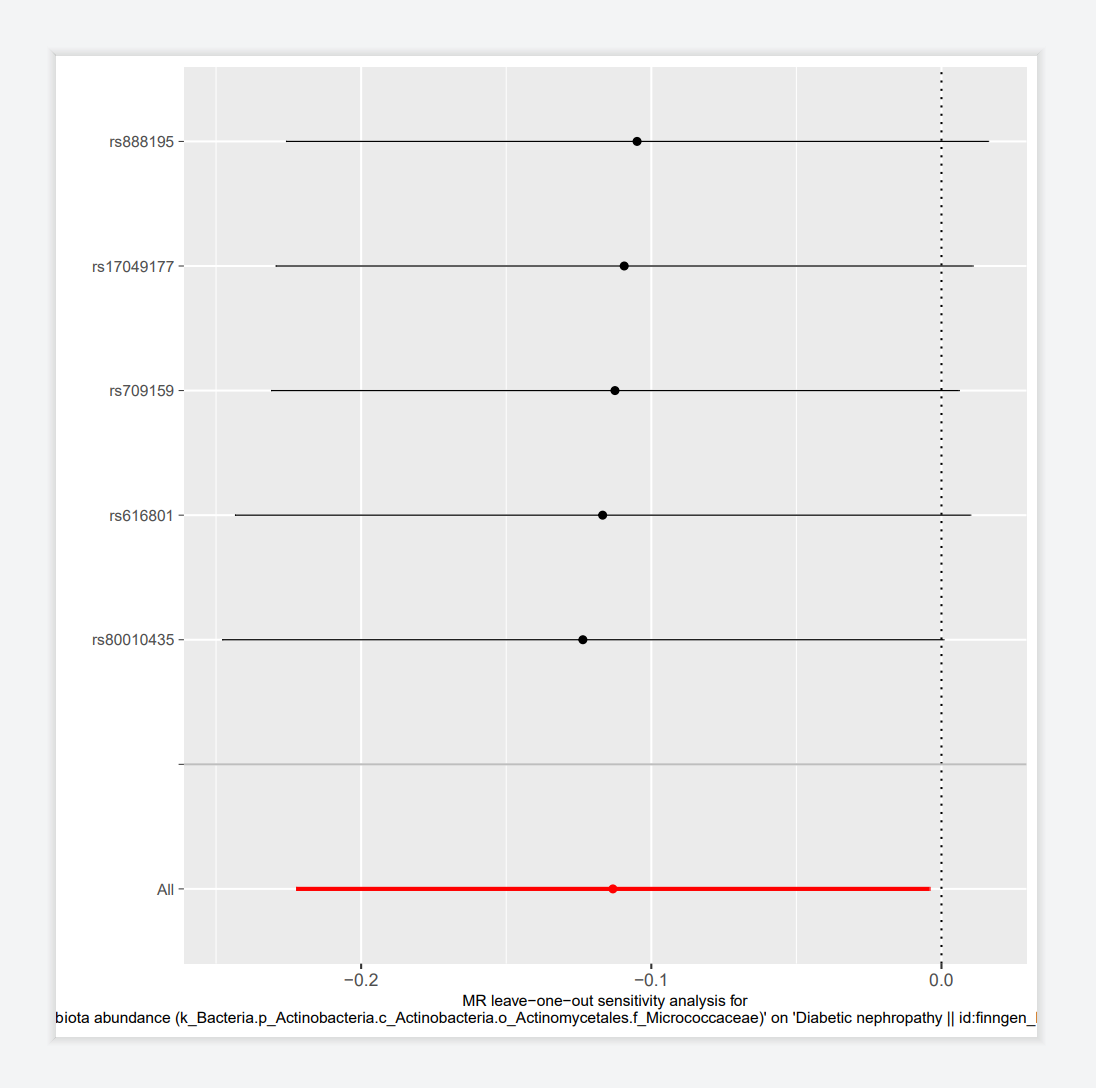

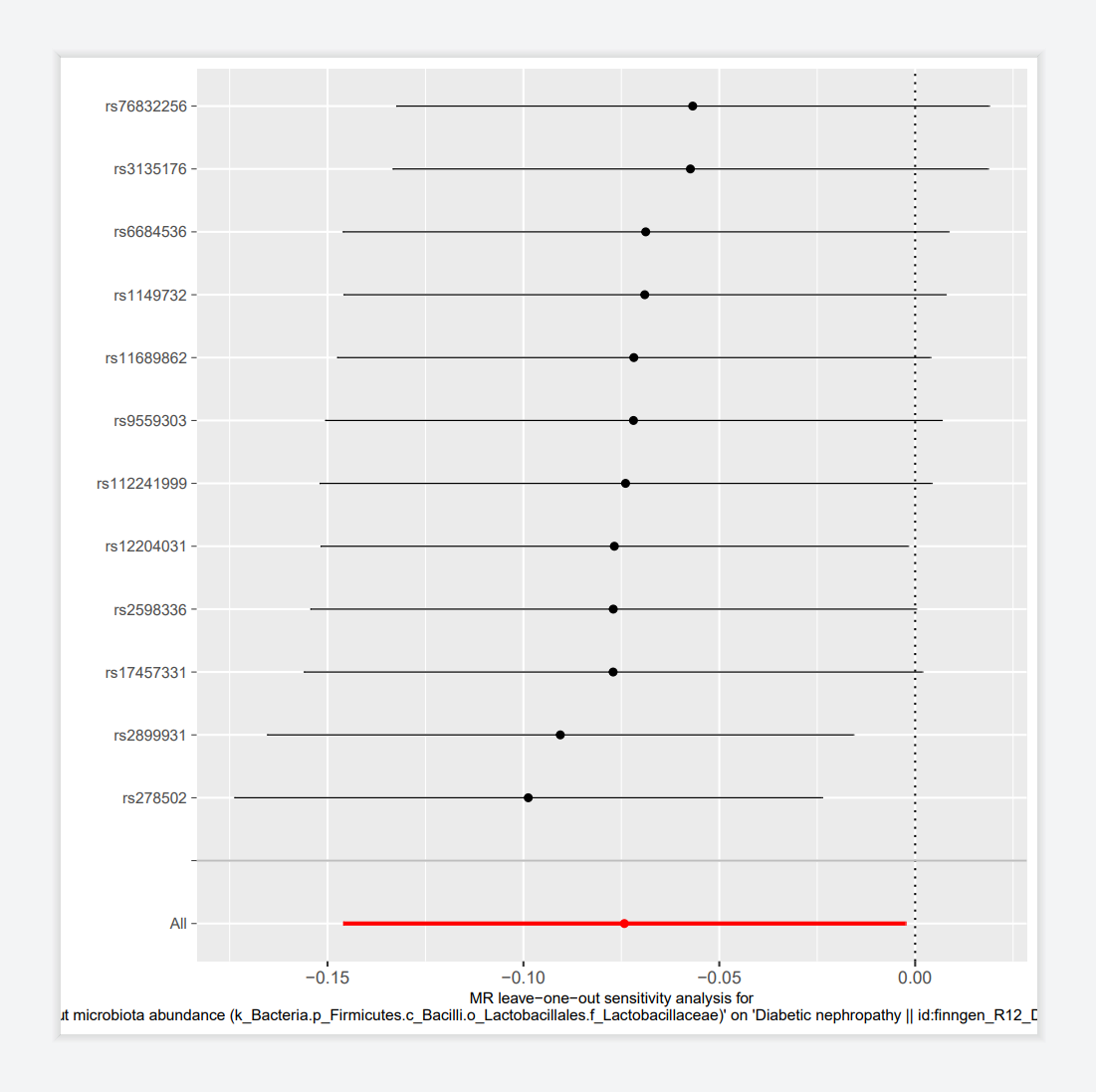


**C D**

**
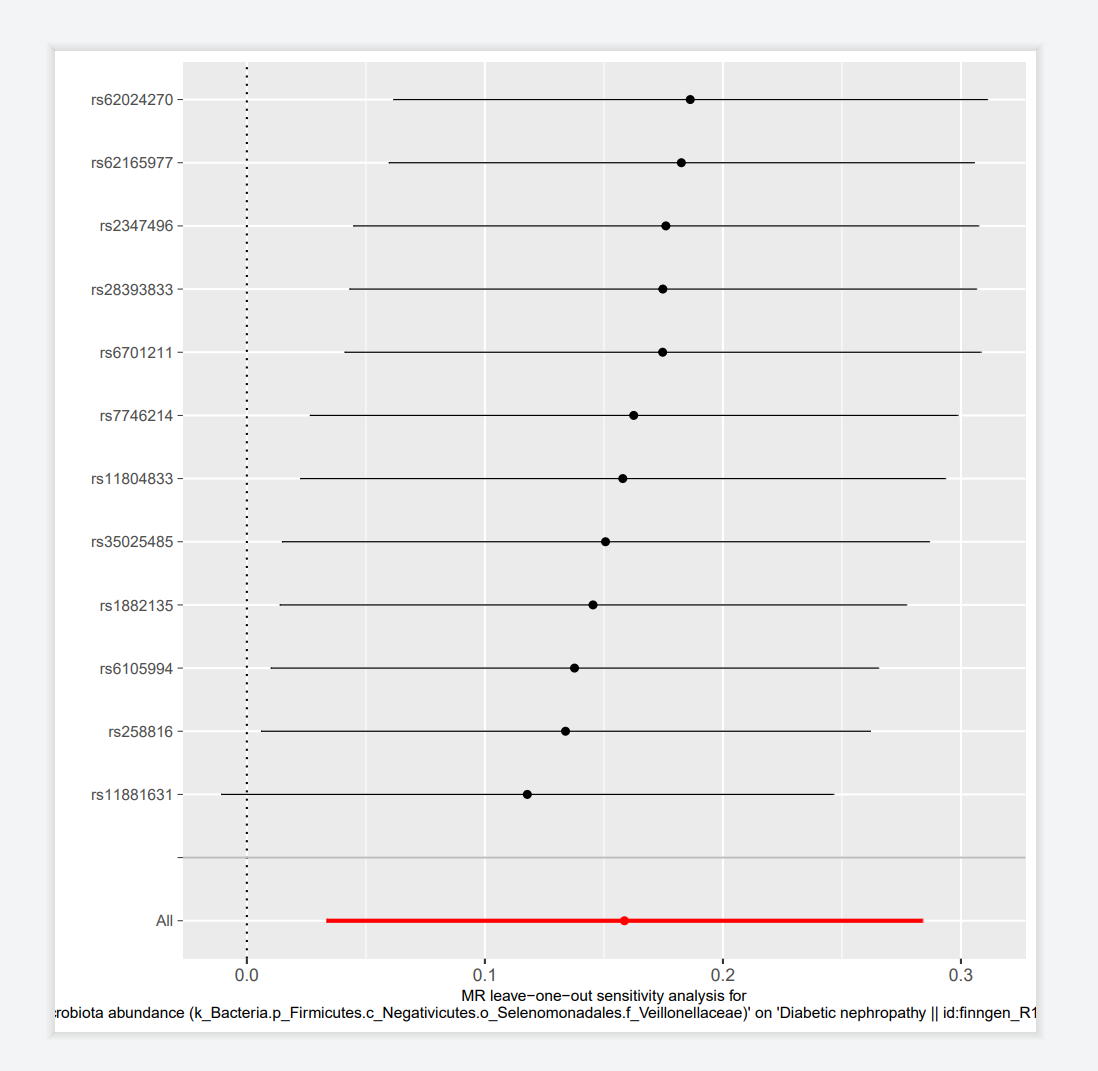

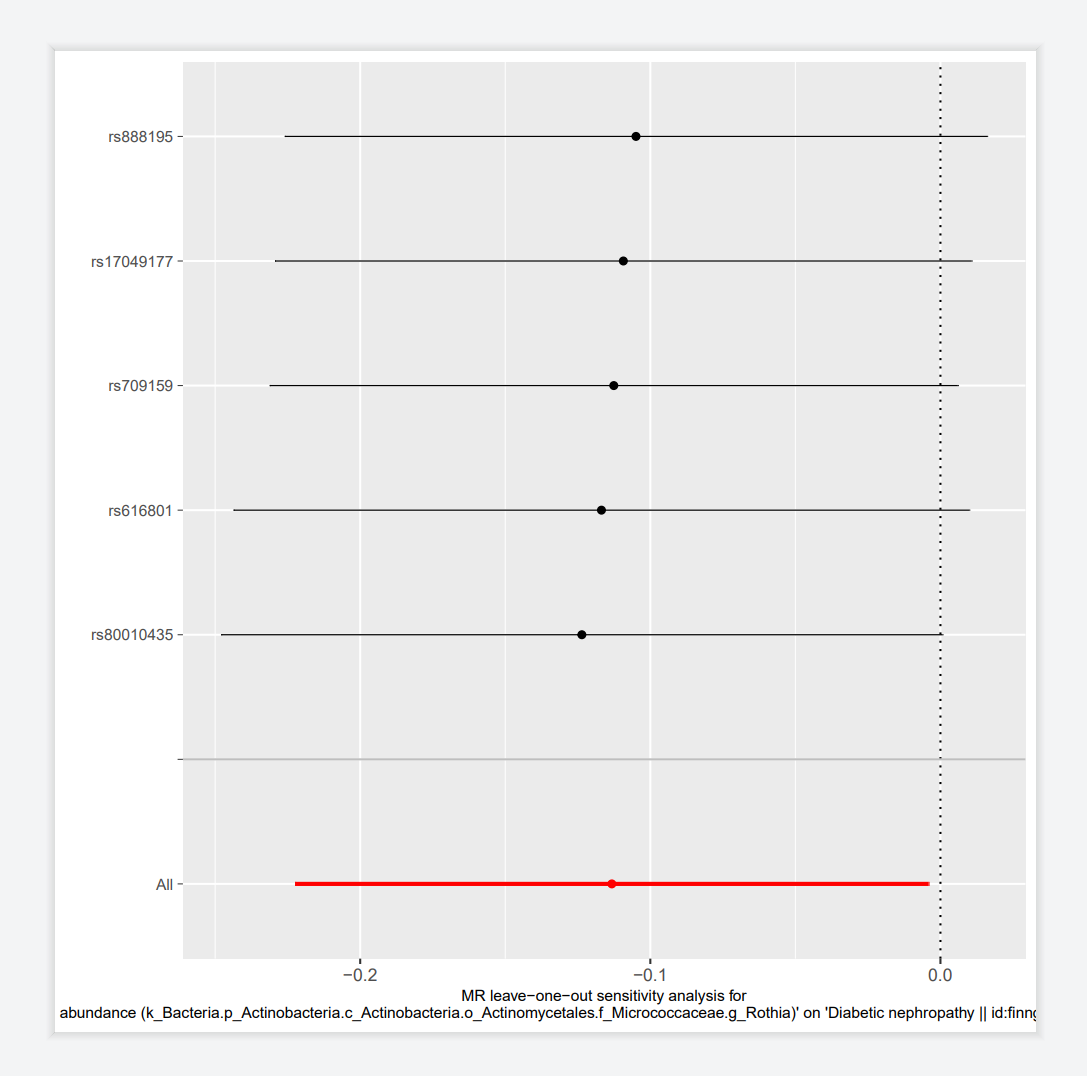
**

**E F**

**
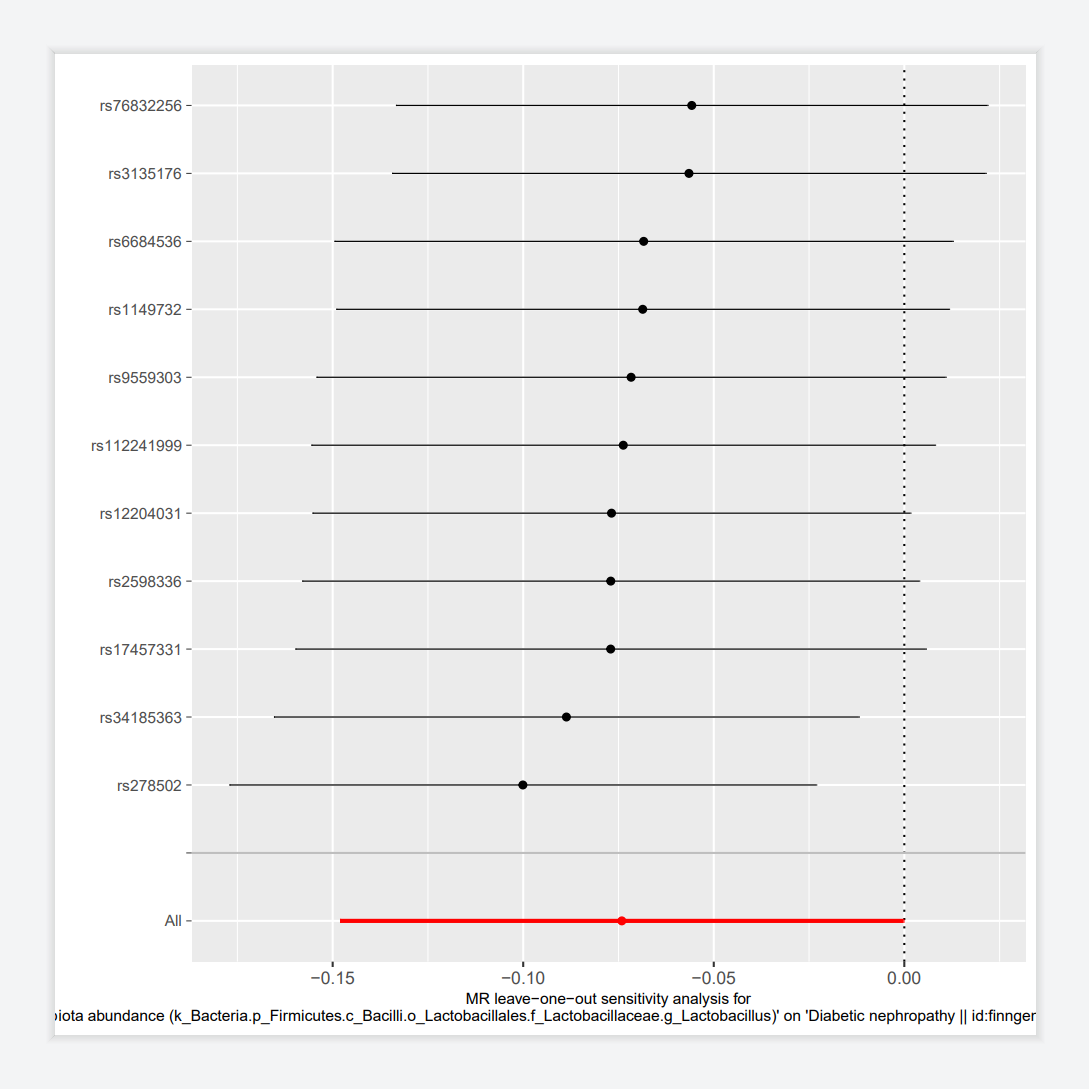

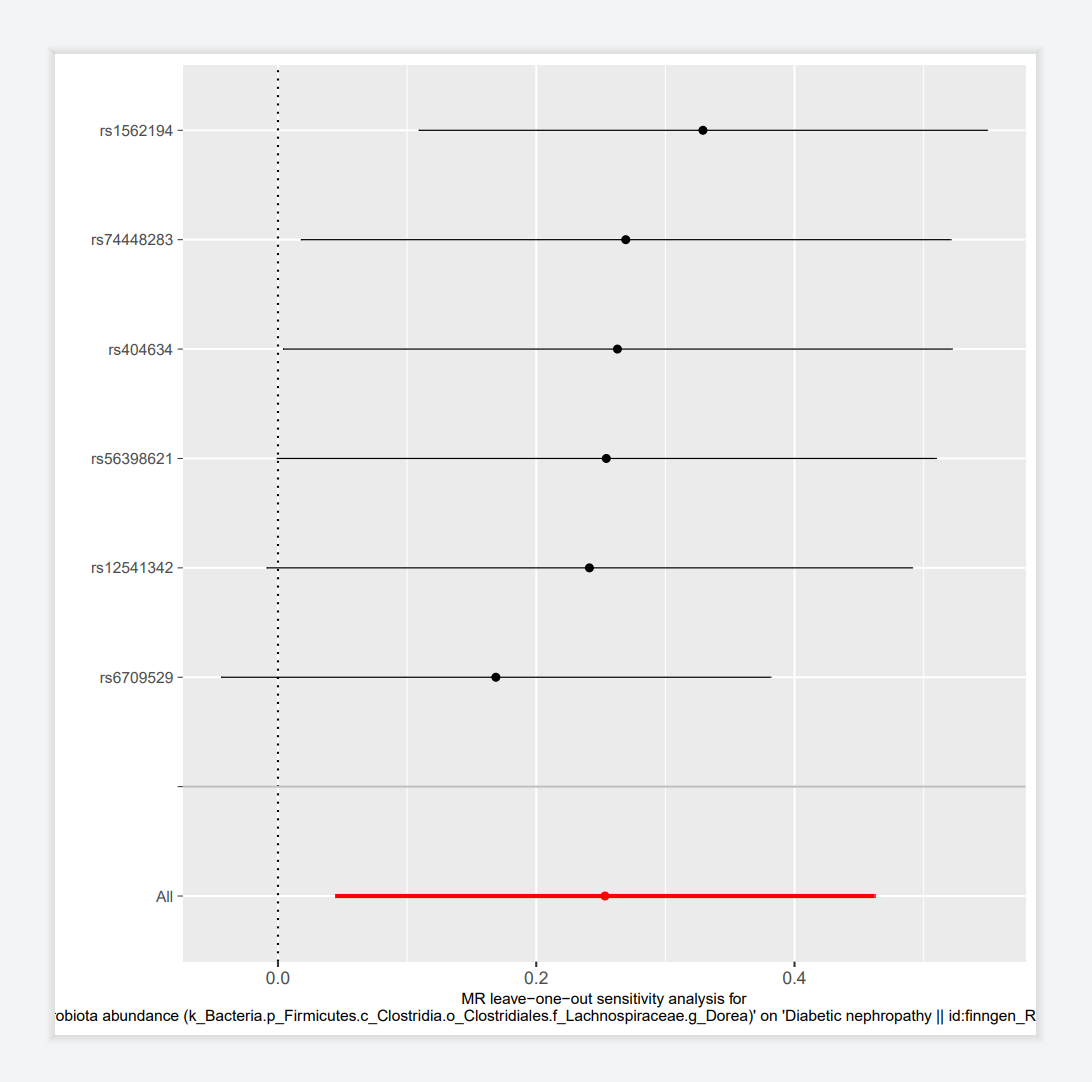
**

**G H**

**
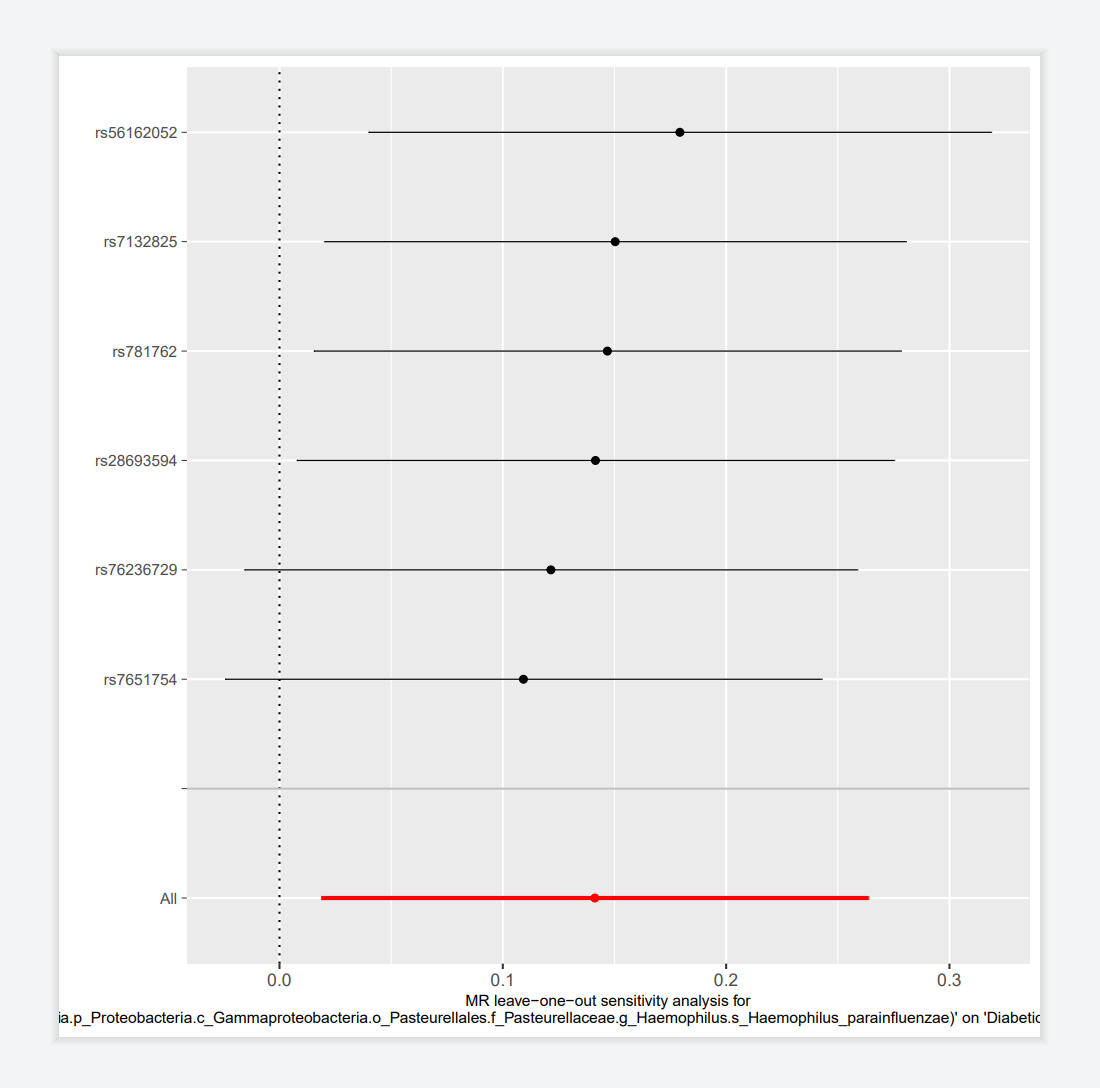

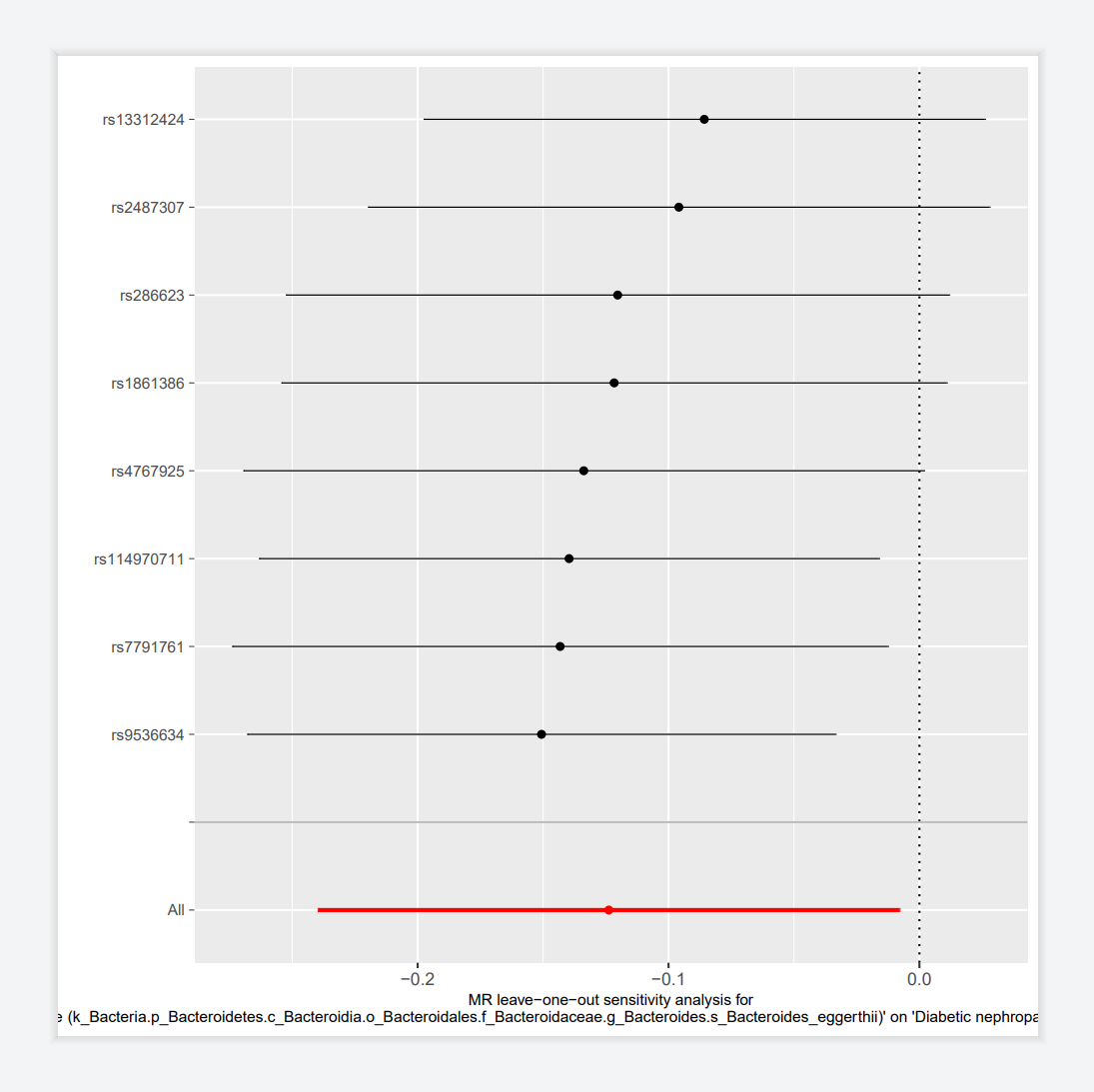
**

**Figure S1.** Leave-one-out analysis for gut microbiota on diabetic nephropathy. The analyses of (A) f_Micrococcaceae, (B) f_Lactobacillaceae, (C) f_Veillonellaceae, (D) g_Rothia, (E) g_Lactobacillus, (F) g_Dorea, (G) s_Haemophilus_parainfluenzae, and(H) s_Bacteroides_eggerthii.

**A B**

**
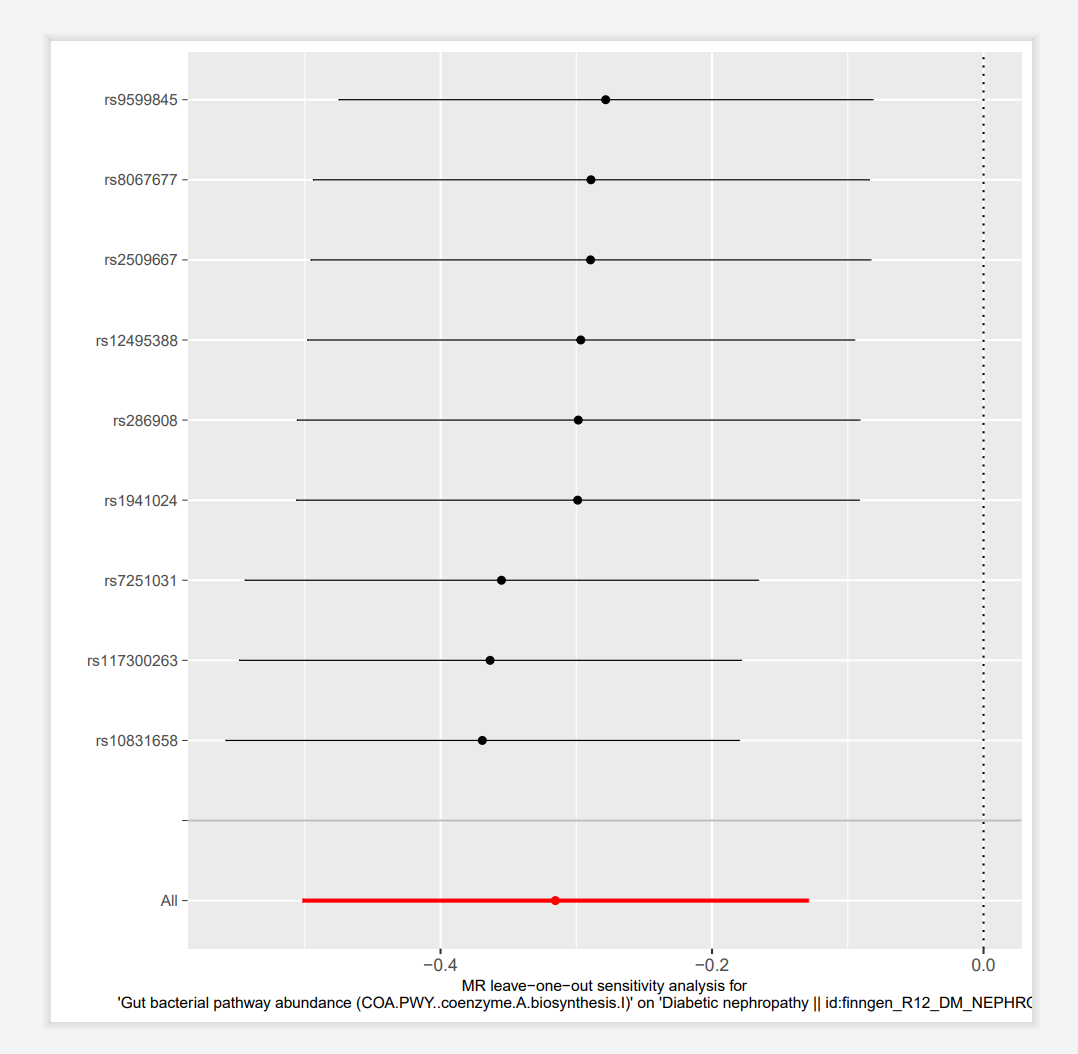

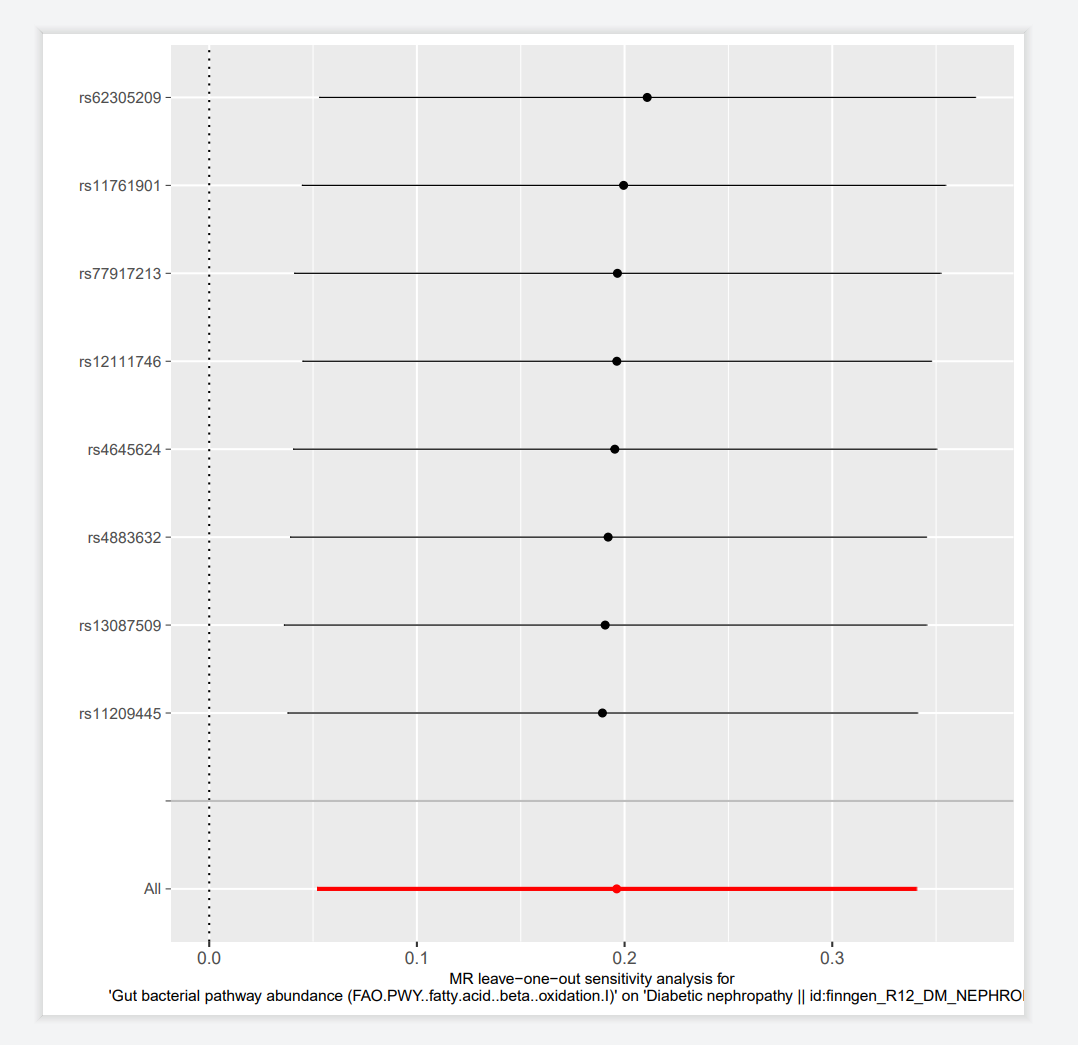
**

**C D**


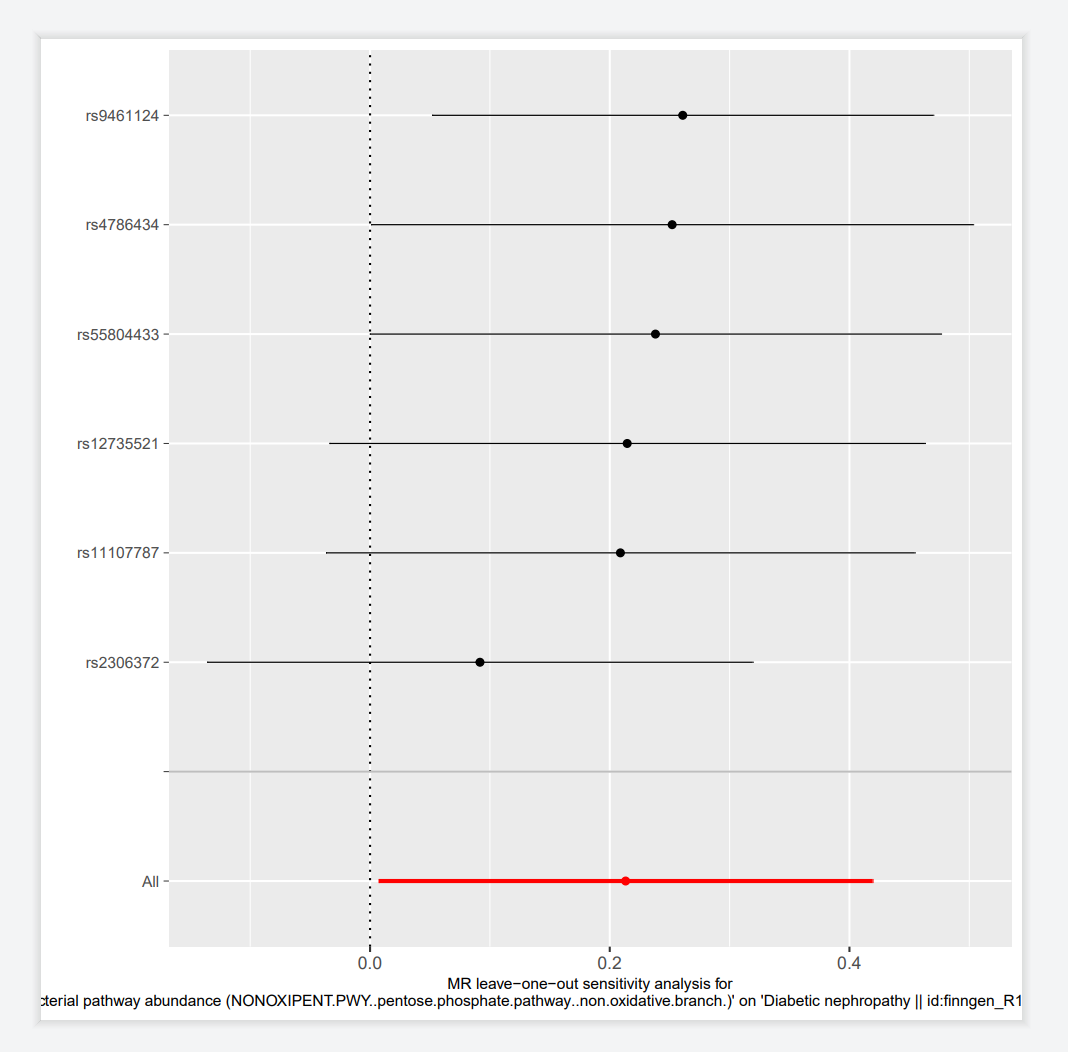

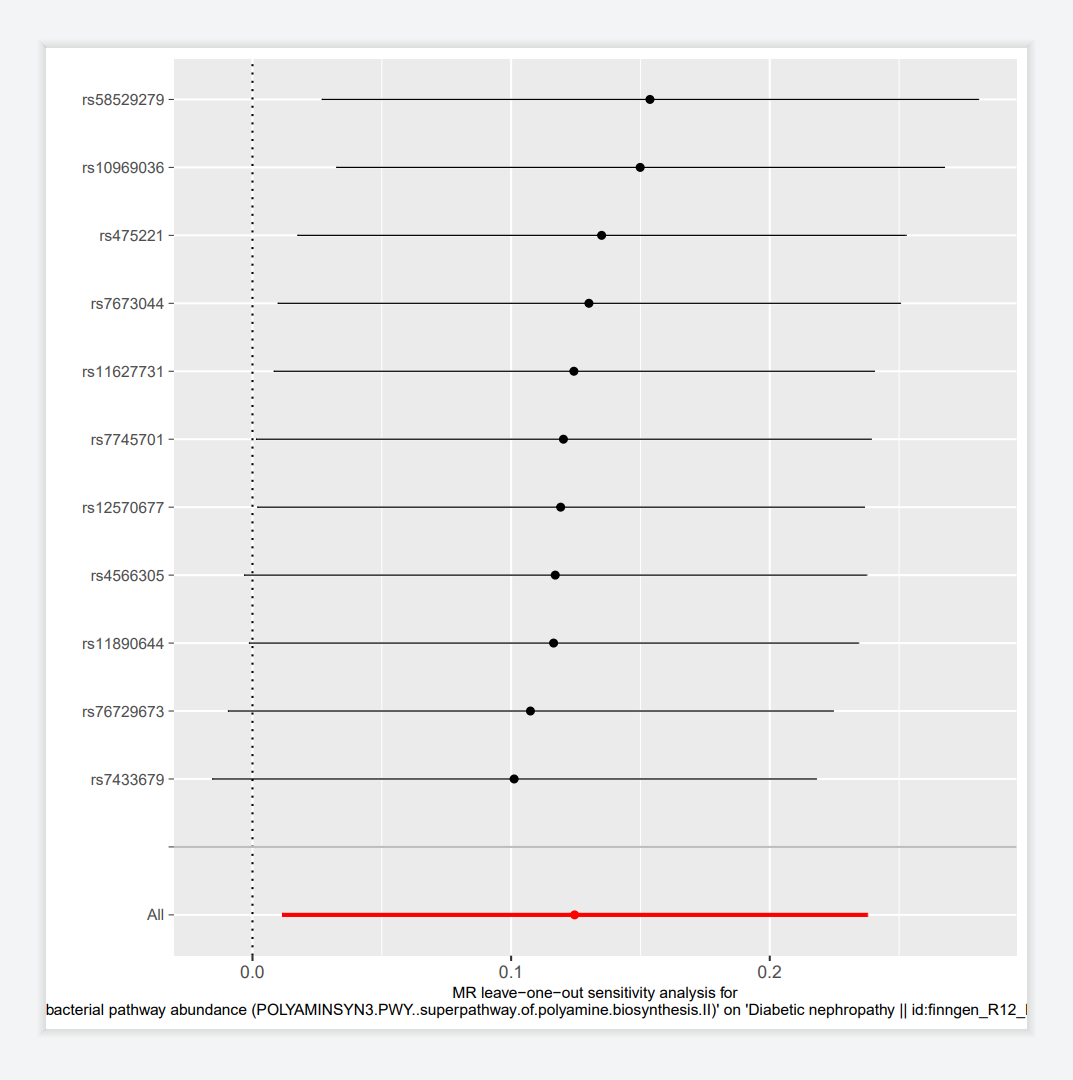


**E F**


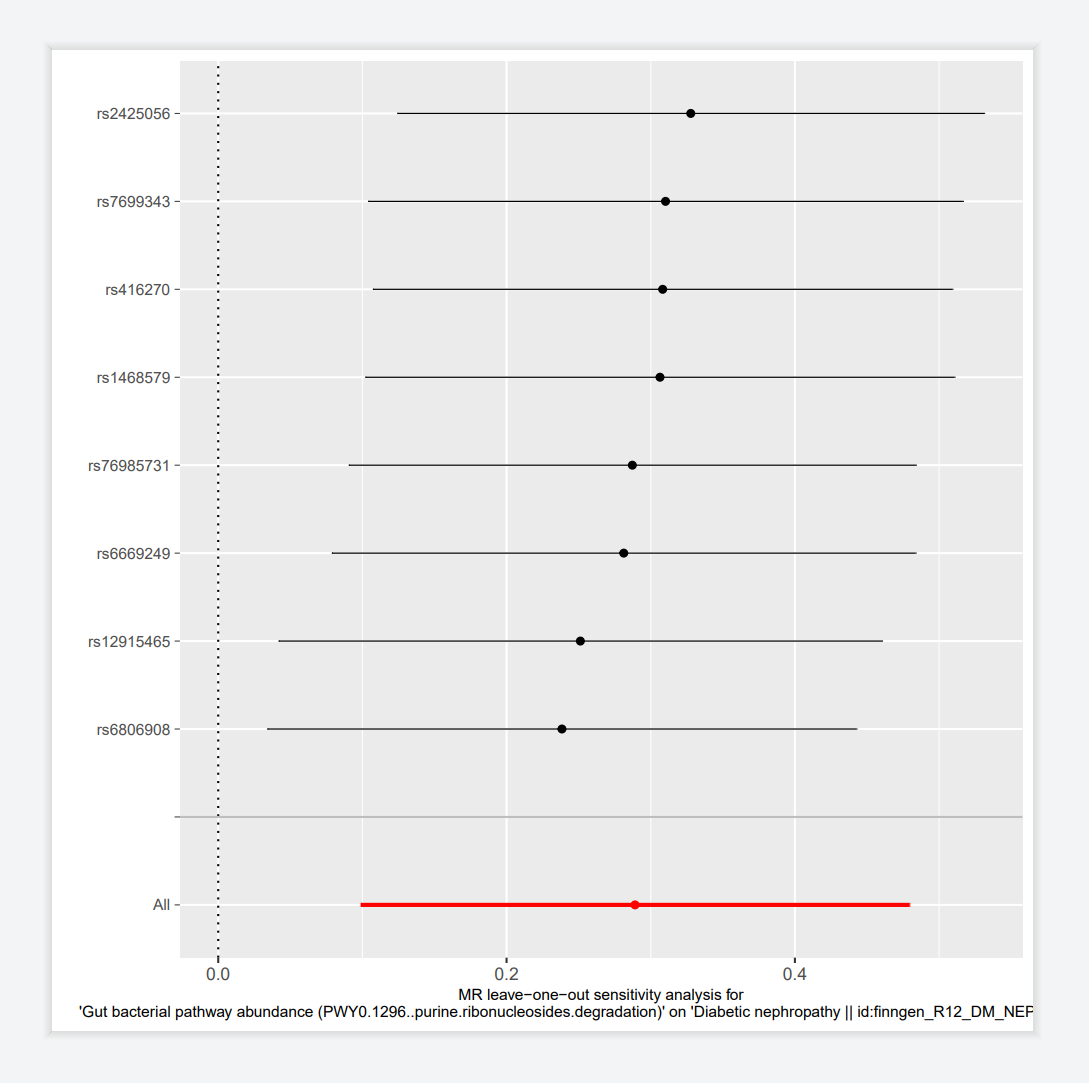

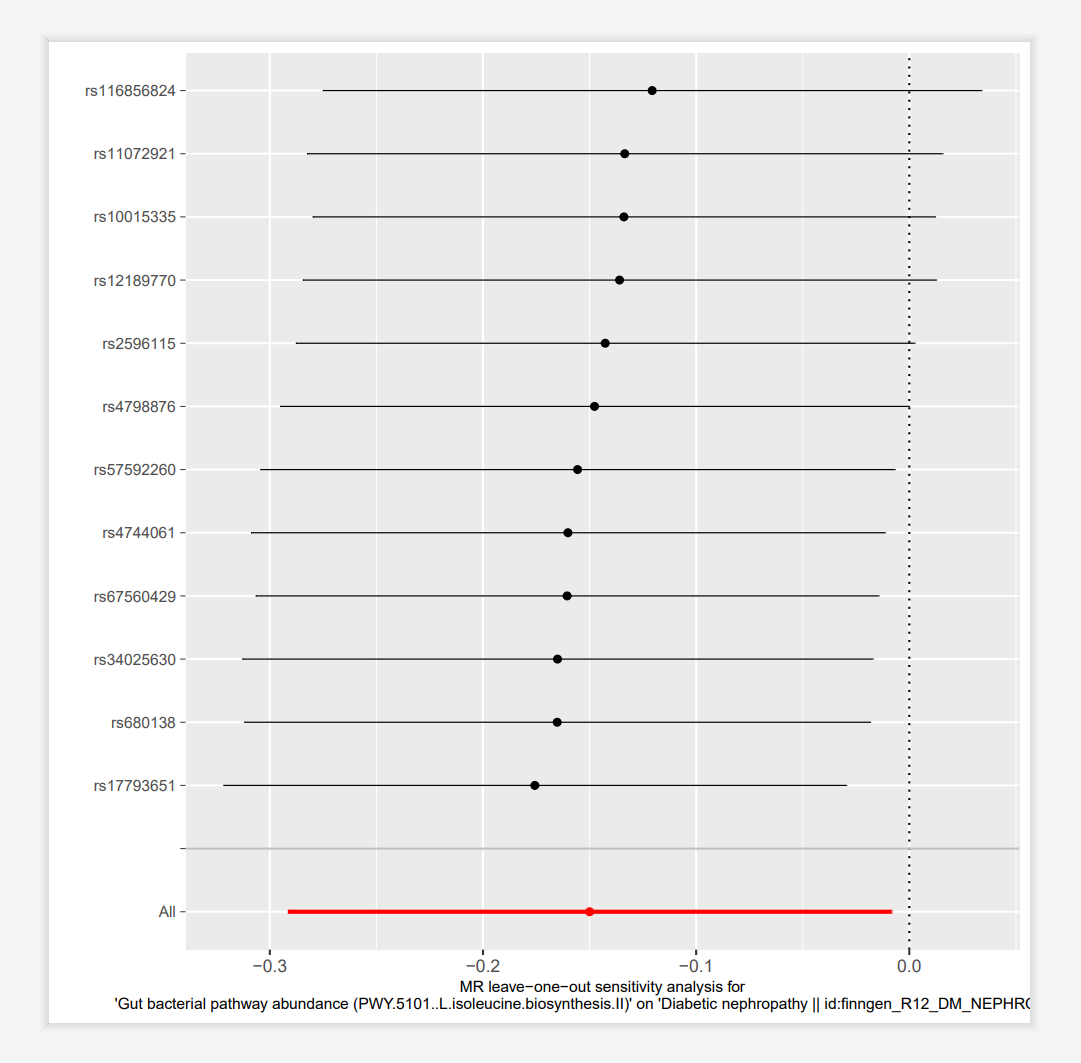


**G**

**
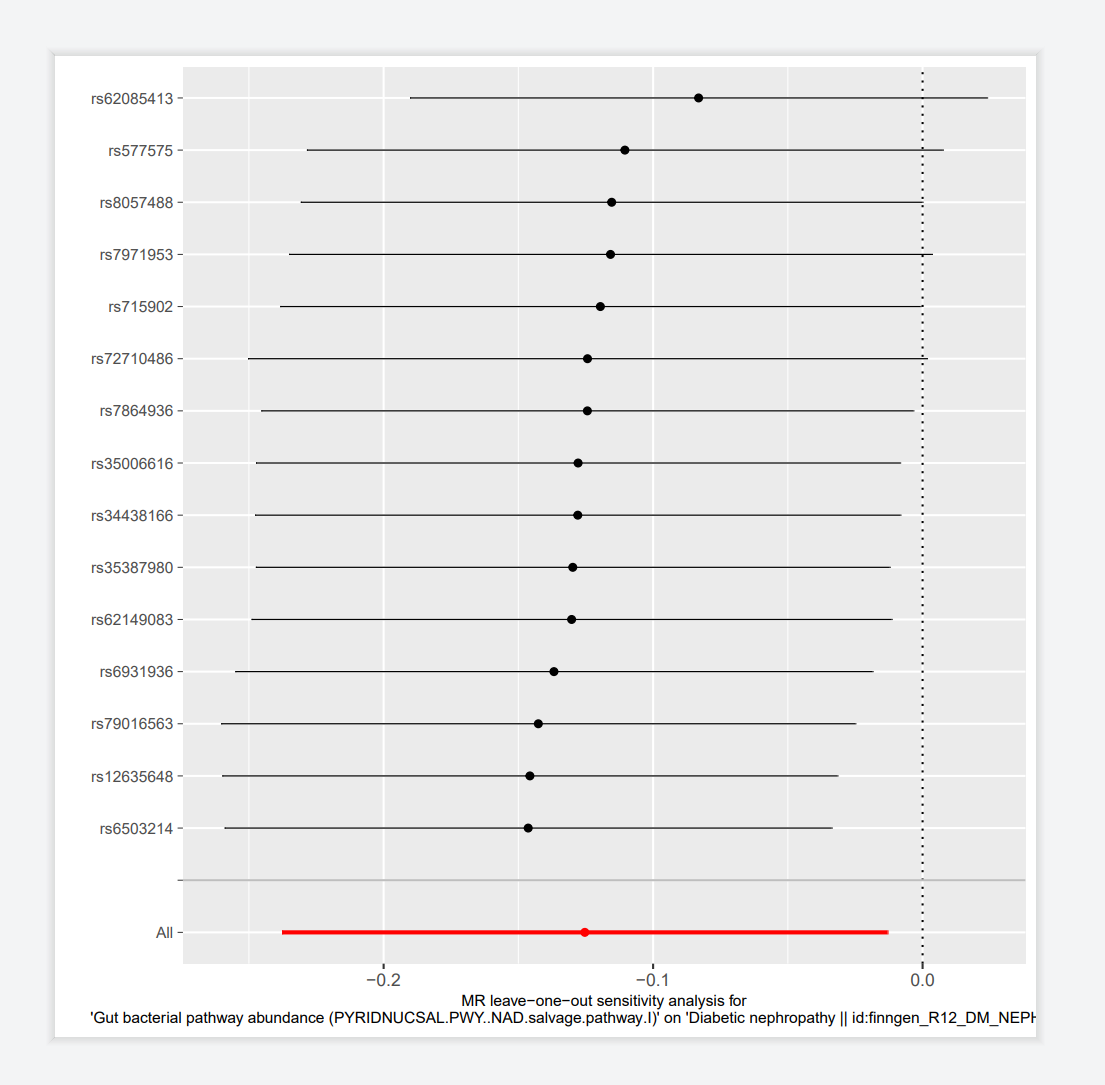
**

**Figure S2.** Leave-one-out analysis for gut bacterial pathway abundances (GBPAs) on diabetic nephropathy. The analyses of

(A) GBPA_ COA.PWY..coenzyme.A.biosynthesis.I,

(B) GBPA_FAO.PWY..fatty.acid..beta..oxidation.I,

(C) GBPA_NONOXIPENT.PWY..pentose.phosphate.pathway..non.oxidative.branch., (D) GBPA_POLYAMINSYN3.PWY..superpathway.of.polyamine.biosynthesis.II,

(E) GBPA_PWY0.1296..purine.ribonucleosides.degradation,

(F) GBPA_PWY.5101..L.isoleucine.biosynthesis.II,

and(G) GBPA_PYRIDNUCSAL.PWY..NAD.salvage.pathway.I.
